# Facile synthesis of a 3-deazaadenosine phosphoramidite for RNA solid-phase synthesis

**DOI:** 10.3762/bjoc.12.250

**Published:** 2016-11-28

**Authors:** Elisabeth Mairhofer, Elisabeth Fuchs, Ronald Micura

**Affiliations:** 1 Institute of Organic Chemistry and Center for Molecular Biosciences, University of Innsbruck, Austria

**Keywords:** deazapurine nucleoside, nucleosidation, protection groups, ribozymes

## Abstract

Access to 3-deazaadenosine (c^3^A) building blocks for RNA solid-phase synthesis represents a severe bottleneck in modern RNA research, in particular for atomic mutagenesis experiments to explore mechanistic aspects of ribozyme catalysis. Here, we report the 5-step synthesis of a c^3^A phosphoramidite from cost-affordable starting materials. The key reaction is a silyl-Hilbert–Johnson nucleosidation using unprotected 6-amino-3-deazapurine and benzoyl-protected 1-*O*-acetylribose. The novel path is superior to previously described syntheses in terms of efficacy and ease of laboratory handling.

## Introduction

The synthesis of 3-deazaadenosine building blocks for RNA solid-phase synthesis represents a severe bottleneck in modern RNA research, in particular for studies that aim at the mechanistic elucidation of site-specific backbone cleavage of recently discovered ribozyme classes, known as twister, twister sister, pistol, and hatchet RNA motives [1–2]. Selected adenines in their active sites have been discussed to participate in acid base catalysis, thereby contributing to accelerate the specific phosphodiester cleavage of these nucleolytic ribozymes. Concerning the twister ribozyme, structural analyses suggest that an adenine N3 atom plays a dominant role in catalysis [3–5]. Also for the pistol ribozyme, evidence exists that an adenine-N3 in the active site is significant for the cleavage activity, most likely by 5’-*O*-leaving group stabilization through proton shuttling [6–7]. Another example for a specific role of an adenine-N3 is associated with the catalysis during ribosomal peptide bond formation, a proposal about its role in proton transfer has been disputed heavily since the first ribosome crystal structures up to very recent investigations [8–10]. The involvement of N3, and not N1, is surprising with respect to basicity of these purine nitrogen atoms, because N1 represents the major protonation site, followed by N7 and N3. This order is deduced from the macroscopic p*K*_a_ values that were measured for adenine, 9-methyladenine, and adenosine [11]. Importantly, there is growing evidence that the p*K*_a_ values of nucleobases can be significantly shifted within a well-structured RNA fold [12–15].

To address RNA phenomena of that kind, comparative atomic mutagenesis is an indispensable means, and with respect to ribozymes, can deliver important insights into the RNA catalyzed chemical reactions and underlying mechanisms. Therefore, 1-deazaadenosine (c^1^A), 1-deaza-2’-deoxyadenosine (c^1^dA), 3-deazaadenosine (c^3^A), and 3-deaza-2’-deoxyadenosine (c^3^dA), and the corresponding phosphoramidites to prepare oligoribonucleotides are highly requested nucleoside modifications. Unfortunately, synthetic approaches to achieve them are troublesome and time consuming, in particular for c^3^A. To the best of our knowledge, only two papers have reported the synthesis of c^3^A phosphoramidites so far [16–17]. Thereby, the major bottleneck is access to the naked nucleoside. Although the c^3^A nucleoside is commercially available, prices in the hundreds of Euro range for low milligram amounts make this source unsatisfying. The previously reported c^3^A phosphoramidite synthesis from our laboratory [16], which took older reports by Matsuda, Piccialli, McLaughlin, Watanabe, Robins, and co-workers into account [17–21], started from inosine leading to c^3^A after 8 steps via a 5-amino-4-imidazolecarboxamide (AICA) riboside derivative with 8% overall yield. Another 4 steps followed to achieve a properly protected building block for RNA solid-phase synthesis [16]. With a total of 12 steps, the approach is not very attractive. Because of this frustrating situation, we set out to develop an efficient and easy-to-handle synthesis of a 3-deazaadenosine phosphoramidite building block.

## Results and Discussion

### Previously described synthetic routes to c^3^A via nucleosidation

**Scheme 1:**
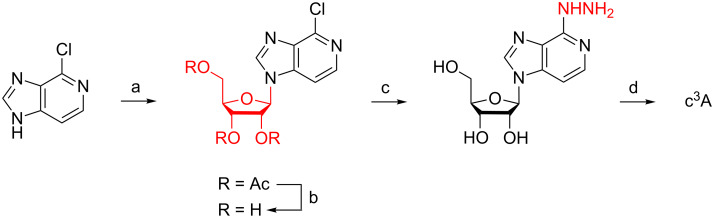
Synthesis of c^3^A described by Rousseau et al. in 1966 [22]. a) 1,2,3,5-Tetraacetyl-ß-D-ribofuranose, chloroacetic acid (cat.), 175 °C (melting until clear solution). b) NH_3_ in CH_3_OH, 0 °C, 14 h. c) Anhydrous hydrazine, steam bath, 1 h, not isolated. d) Raney nickel, water, reflux, 1 h.

In 1966, Rousseau, Townsend, and Robins reported the nucleosidation of 4-chloroimidazo[4,5-*c*]pyridine and 1,2,3,5-tetraacetyl-ß-D-ribofuranose in the presence of chloro acetic acid to yield the corresponding 6-chloro-3-deazapurine nucleoside (Scheme 1) [22]. Subsequent attempts to convert the chlorine atom directly by amination under various conditions failed. Only when treated with hydrazine, nucleophilic substitution was observed and after reduction with Raney nickel the desired 3-deazaadenosine was isolated. Our own attempts towards direct ammonolysis failed as well. Additionally, the limited commercial availability of hydrazine and its inconvenience in handling excluded this route for our purposes.

**Scheme 2:**
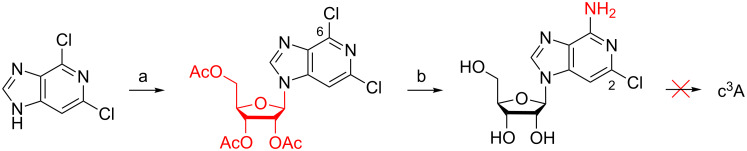
Synthesis of c^3^A described by Montgomery et al. in 1977 [23]. The final step, displacement of the 2-chlorine atom by a hydrogen atom, remains problematic [24–26]. a) 1,2,3,5-Tetraacetyl-ß-D-ribofuranose, *p*-toluenesulfonic acid (cat.), melt (160 °C), 5 to 10 min. b) NH_3_ in ethanol (saturated at −30 °C), 140 °C, 89 h [23] or NH_3_ (30% aq), 200 °C, 5 d, 80% [24].

In 1977, Montgomery, Shortnacy, and Clayton, reported the preparation of 6-chloro-3-deazapurine ribonucleoside via nucleosidation of 4,6-dichloroimidazo[4,5-*c*]pyridine with 1,2,3,5-tetraacetyl-ß-D-ribofuranose in the presence of *p*-toluenesulfonic acid (Scheme 2) [23–24]. Treatment of the 2,6-dichloro-3-deazapurine derivative with ammonia was optimized by Bande et al. recently [25], but still required 200 °C reaction temperature and five days reaction time to afford regioselective displacement of the 2-chlorine atom and concomitant deacetylation in high yield. Unfortunately, all attempts of the authors to displace the second chlorine atom of the imidazo[4,5-*c*]pyridine nucleoside using sodium methoxide or palladium-catalyzed cross-coupling reactions as described in [26] failed. We therefore decided not to put additional efforts into this route.

### Attempts to use 6-azido-3-deazapurine ribonucleoside as key intermediate

**Scheme 3:**
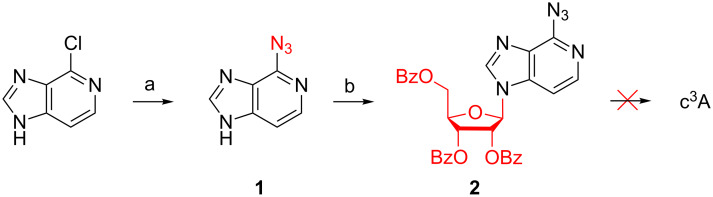
Synthesis of tribenzoylated 6-azido-3-deazapurine nucleoside **2**. a) LiN_3_ (1.3 equiv), *N*,*N*-dimethylformamide, 120 °C, 18 h, 76%. b) 1-*O*-Acetyl-2,3,5-tri-*O*-benzoyl-ß-D-ribofuranose (1 equiv), *N*,*O*-bis(trimethylsilyl)acetamide (3.4 equiv), trimethylsilyl trifluoromethanesulfonate (3.0 equiv), 105 °C, 70 min, 71%.

Our initial attempts to create an efficient route to c^3^A started with the smooth transformation of commercially available 4-chloroimidazo[4,5-*c*]pyridine with lithium azide to provide 4-azidoimidazo[4,5-*c*]pyridine (**1**) [27] (Scheme 3). Then, glycosylation with 1-*O*-acetyl-2,3,5-tri-*O*-benzoyl-ß-D-ribofuranose gave the desired nucleoside **2** in high yield. Unfortunately, all our attempts to find appropriate conditions to reduce the 6-azido group to the corresponding amine failed. In short, these trials included i) hydrogenation under Pd/C catalysis at elevated pressure (30 psi) in ethanol or *N*,*N*-dimethylacetamide, ii) ammonium formiate, Pd/C, in methanol [28], iii) tin(II) chloride, in ethanol [29], iv) thioacetic acid, lutidine, in CH_2_Cl_2_ [30], v) triphenylphosphine, in CH_2_Cl_2_, aqueous work-up, and finally vi) Mg^0^ in methanol.

### Efficient 5-step synthesis of 3-deazaadenosine phosphoramidite

**Scheme 4:**
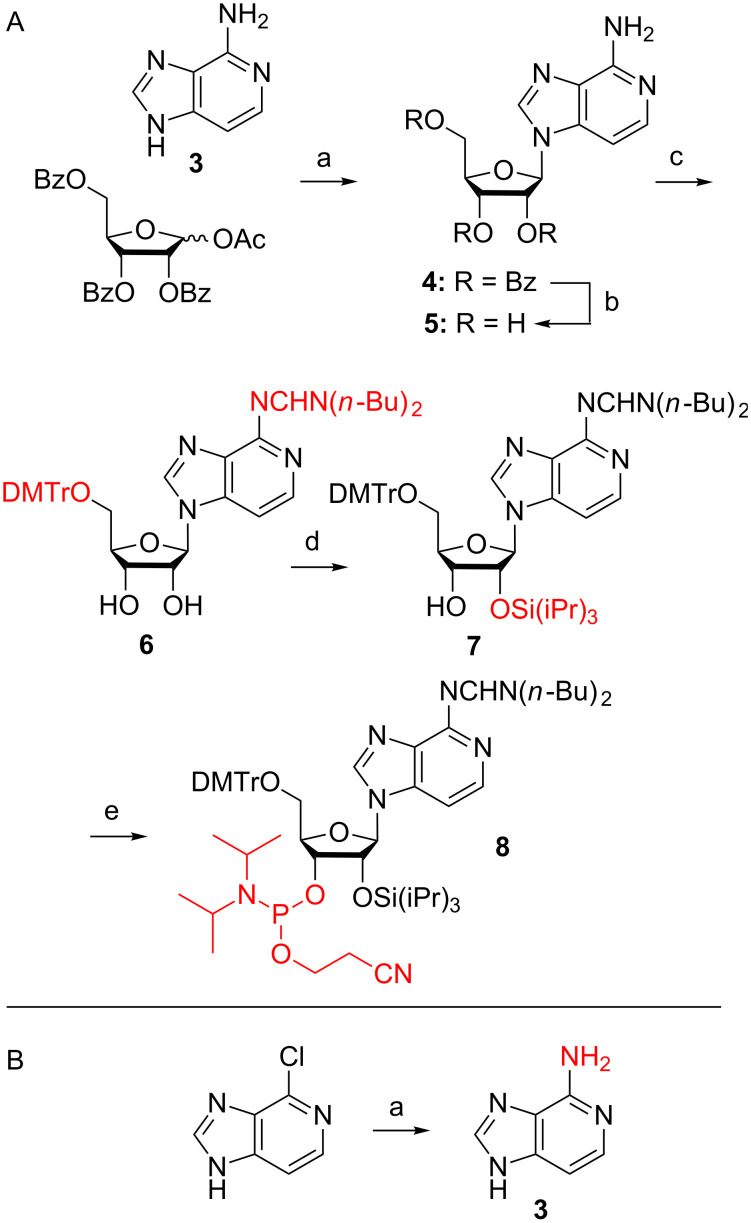
Efficient 5-step synthesis of 3-deazaadenosine phosphoramidite **8** from commercially available, affordable starting materials. (A) Reagents and conditions: a) *N*,*O*-bis(trimethylsilyl)acetamide (3.5 equiv), trimethylsilyl trifluoromethanesulfonate (3.0 equiv), in toluene, 105 °C, 3.5 h, 63%. b) CH_3_NH_2_, in water/ethanol, room temperature, 18 h, 95%. c) i) *N*,*N*-Dibutylformamide dimethyl acetal (2.3 equiv), in pyridine, ii) 4,4’-dimethoxytrityl chloride (2.7 equiv), 4-(dimethylamino)pyridine (0.3 equiv), in pyridine, room temperature, 16 h, 40%. d) Triisopropylsilyl chloride (4 equiv), AgNO_3_ (4 equiv), 18 h, 26%. e) 2-Cyanoethyl *N*,*N*-diisopropylchlorophosphoramidite (3 equiv), *N,N-*dimethylethylamine (10 equiv), in dichloromethane, room temperature, 105 min, 44%. (B) Reagents and conditions: a) aqueous ammonia, microwave, 140 °C, 2 h, 90%.

The key step of our novel route to c^3^A phosphoramidite (Scheme 4) is a silyl-Hilbert–Johnson nucleosidation reaction of commercially available 4-aminoimidazo[4,5-*c*]pyridine (**3**) and 1-*O*-acetyl-2,3,5-tri-*O*-benzoyl-ß-D-ribofuranose in the presence of *N*,*O*-bis(trimethylsilyl)acetamide and trimethylsilyl trifluoromethanesulfonate in toluene. No protection of the 4-amino group of compound **3** was required. The reaction proceeded in high yields and gave the tribenzoylated c^3^A nucleoside **4**. This compound was analysed by ^1^H ROESY NMR spectroscopy which was consistent with the structure of the desired ß-N9 isomer **4**, indicated by strong ROEs of the nucleobase C3-H with ribose C3’-H, C2’-H and C1’-H (see Supporting Information File 1). The benzoyl groups of nucleoside **4** were then cleaved with methylamine in ethanol and water to furnish the free c^3^A nucleoside **5**. An authentic reference sample that was synthesized according to the previously established 12-step route was used for direct spectroscopic comparison (see Supporting Information File 1) and additionally confirmed its identity. Then, treatment with *N*,*N*-dibutylformamide dimethyl acetal [31] resulted in amidine protection of the exocyclic C6-NH_2_ group. At the same time, the applied excess of the reagent allowed to transiently form the corresponding nucleoside 2’,3’-*O*-acetal [32], leaving the primary 5’-OH group available for selective tritylation with 4,4’-dimethoxytrityl chloride to give compound **6**. Selective protection of the 2’-OH was challenging. Initial attempts that focused on the introduction of the TBDMS group according to the procedure described by McLaughlin and co-workers [17] were unsuccessful. Also, attempts to introduce the [(triisopropylsilyl)oxy]methyl group (TOM) following standard procedures [32] unfortunately failed. We encountered these problems already in our previously published synthesis for *N*^6^-benzoyl protected c^3^A phosphoramidite [16], and therefore, we decided to apply triisopropylsilyl chloride (TIPS-Cl) and silver nitrate which resulted in the desired 2’-*O*-TIPS protected nucleoside **7** in 28% yield after chromatographic separation from the corresponding 3’-regioisomer. Finally, the 5’-*O*-DMTr-2’-*O*-TIPS protected 3-deazaadenosine derivative **7** was converted into the phosphoramidite building block **8** with 2-cyanoethyl diisopropylchlorophosphoramidite in the presence of *N*-dimethylethylamine. Starting from compound **3**, our route provides **8** in a 6% overall yield in five steps with four chromatographic purifications; in total, 0.6 g of **8** was obtained in the course of this study.

## Conclusion

With the reported 5-step synthesis of a c^3^A phosphoramidite we created a route that is superior to previously described syntheses in terms of efficacy and ease of laboratory handling. The key reaction is a silyl-Hilbert–Johnson nucleosidation using unprotected 6-amino-3-deazapurine and benzoyl-protected 1-*O*-acetylribose, providing 3-deazaadenosine (c^3^A) in high yields for the subsequent functionalizations to yield a properly protected building block for RNA solid-phase synthesis.

The so-obtained c^3^A-modified RNAs are currently used for atomic mutagenesis experiments to explore mechanistic aspects of phophodiester cleavage of recently discovered ribozyme classes, such as twister, pistol, and hatchet ribozymes [1–2,33].

## Experimental

**General.** Chemical reagents and solvents were purchased from commercial suppliers (Sigma-Aldrich) and used without further purification. 4-Aminoimidazo[4,5-*c*]pyridine (6-amino-3-deazapurine) and 4-chloroimidazo[4,5-*c*]pyridine (6-chloro-3-deazapurine) were purchased from Synthonix and Carbogen. Organic solvents for reactions were dried overnight over freshly activated molecular sieves (4 Å). The reactions were carried out under an argon atmosphere. Analytical thin-layer chromatography (TLC) was carried out on Marchery-Nagel Polygram SIL G/UV254 plates. Column chromatography was carried out on silica gel 60 (70–230 mesh). ^1^H, and ^13^C NMR spectra were recorded on Bruker DRX 300 MHz and Bruker Avance II+ 600 MHz instruments. Chemical shifts (δ) are reported relative to tetramethylsilane (TMS) and referenced to the residual proton or carbon signal of the deuterated solvent: CDCl_3_ (7.26 ppm) or DMSO-*d*_6_ (2.49 ppm) for ^1^H NMR; CDCl_3_ (77.0 ppm) or DMSO-*d*_6_ (39.5 ppm) for ^13^C NMR spectra. ^1^H and ^13^C assignments are based on COSY and HSQC experiments. MS experiments were performed on a Waters ESI TOF LCT Premier Serie KD172 or Bruker 7T FT-ICR instrument with an electrospray ion source. Samples were analyzed in the positive-ion mode.

**2',3',5'-Tri-*O*-benzoyl-3-deazaadenosine (4)*.*** 1-*O*-Acetyl-2,3,5-tri-*O*-benzoyl-β-D-ribofuranose (1.1360 g, 2.25 mmol) and compound **3** (303.6 mg, 2.26 mmol) were suspended in dry toluene (50 mL) under an argon atmosphere, after which *N*,*O*-bis(trimethylsilyl)acetamide (1.92 mL, 7.85 mmol) was added at room temperature. The suspension was heated up and kept at 105 °C for 2.5 h and then cooled to room temperature, resulting in a clear beige solution. Trimethylsilyl trifluoromethanesulfonate (1.22 mL, 6.72 mmol) was added and the solution was stirred for 1 h at 105 °C, followed by evaporation of all volatiles. The residue was diluted in dichloromethane (20 mL) and washed with saturated sodium bicarbonate solution (3 × 30 mL). The combined organic layers were dried over Na_2_SO_4_ and all volatiles were evaporated. The yellow-beige crude product was purified by flash chromatography (1% methanol in dichloromethane + 1.5% triethylamine, size: 18.0 × 2.5 cm, 23 g silica gel). Yield: 823.7 mg (63%) of compound **4** as beige foam. TLC (10% methanol in dichloromethane): *R*_f_ = 0.52; ^1^H NMR (300 MHz, DMSO-*d*_6_) δ 4.80–4.70 (m, 2H, H-C(5’)), 4.84–4.86 (t, *J* = 3.7 Hz, 1H, H-C(4’)), 5.96–5.99 (t, *J* = 5.3 Hz, 1H, H-C(3’)), 6.03–6.07 (t, *J* = 6.1 Hz, 1H, H-C(2‘)), 6.25 (s, 2H, NH_2_), 6.57–6.59 (d, *J* = 5.9 Hz, 1H, H-C(1’)), 6.93–6.95 (d, *J* = 5.8 Hz, 1H, H-C(3)), 7.39–7.72 (m, 10H, H(benzoyl), H-C(2)), 7.83–7.86 (d, *J* = 7.5 Hz, 2H, H(benzoyl)), 7.97–8.04 (q, *J*_1_ = 13.8 Hz, *J*_2_ = 7.6 Hz, 4H, H(benzoyl)), 8.38 (s, 1H, H-C(8)) ppm; ^13^C NMR (75 MHz, DMSO-*d*_6_) δ 63.67 (C-(5’)), 70.54 (C-(3’)), 72.83 (C-(2’)), 79.37 (C-(4’)), 86.53 (C-(1’)), 96.98 (C-(3)), 125.19, 126.76, 128.08, 128.52, 128.65, 128.69, 128.77 (C(benzoyl)), 129.16, 129.22, 129.29, 129.39 (C(benzoyl)), 133.57, 133.86, 133.95 (C(benzoyl)), 137.12, 139.98 (C-(8)), 140.98 (C-(2)), 152.55, 164.31, 164.70, 165.45 ppm; HRMS [M + H^+^]: calcd for C_32_H_26_N_4_O_7_^+^, 579.1880; found, 579.1852.

**3-Deazaadenosine (5).** Compound **4** (1.231 g, 2.13 mmol) was dissolved in a solution of 33% methylamine in ethanol (10 mL) and 40% methylamine in water (10 mL) and stirred for 18 hours at room temperature. All volatiles were evaporated and the residue was dried in high vacuum. The crude product was purified by dissolving the byproduct (*N*-methylbenzamide) in chloroform and subsequent collection of the precipitate by centrifugation (3000 rpm, rt, 1 min). Yield: 537.1 mg (95%) of compound **5** as beige solid. TLC (30% methanol in dichloromethane): *R*_f_ = 0.23; ^1^H NMR (300 MHz, DMSO-*d*_6_) δ 3.57–3.68 (q, *J* = 10.1 Hz, 2H, H-C(5’)), 3.94–3.96 (d, *J* = 3.3 Hz, 1H, H-C(4’)), 4.08–4.10 (d, *J* = 3.1 Hz, H-C(3’)), 4.30–4.32 (d, *J* = 5.5 Hz, 1H, H-C(2’)), 5.06–5.09 (t, *J* = 6.5 Hz, 1H, OH-C(5’)), 5.19–5.20 (d, *J* = 4.0 Hz, 1H, OH-C(3’)), 5.44–5.46 (d, *J* = 6.2 Hz, 1H, OH-C(2’)), 5.74–5.76 (d, *J* = 6.2 Hz, 1H, H-C(1’)), 6.17 (s, br, 2H, NH_2_), 6.90–6.92 (d, *J* = 5.8 Hz, 1H, H-C(3)), 7.65–7.67 (d, *J* = 5.8 Hz, 1H, H-C(2)), 8.29 (s, 1H, H-C(8)) ppm; ^13^C NMR (75 MHz, DMSO-*d*_6_) δ 61.22 (C-(5’)), 70.08 (C-(3’)), 73.86 (C-(2’)), 85.53 (C-(4’)), 88.66 (C-(1’)), 97.37 (C-(3)), 126.91, 137.58, 139.95 (C-(8)), 140.45 (C-(2)), 152.36 ppm; HRMS [M + H^+^]: calcd for C_11_H_14_N_4_O_4_^+^, 267.1093; found, 267.1074.

***N*^6^-[(Dibutylamino)methylene]-5’-*O*-(4,4’-dimethoxytrityl)-3-deazaadenosine (6).** Compound **5** (199 mg, 747 µmol) was weighed into a 25 mL two-necked flask and dried in high vacuum for 1 hour. Under an argon atmosphere dry pyridine (5 mL) and *N*,*N*-dibutylformamide dimethyl acetal (0.40 mL, 1.74 mmol) were added and stirred for 1.5 h at room temperature. Afterwards, all volatiles were evaporated, the residue dried in high vacuum and redissolved in dry pyridine (5 mL). 4-(Dimethylamino)pyridine (28.4 mg, 233 µmol) and 4,4’-dimethoxytrityl chloride (686 mg, 2.03 mmol) were added and the reaction was allowed to proceed for 16 hours. The reaction was quenched with methanol (1 mL) and all volatiles were evaporated, followed by coevaporation with toluene (2 × 10 mL). The residue was partitioned between dichloromethane (10 mL) and 5% aqueous citric acid solution (7 mL). The organic layer was separated, washed with water and saturated sodium bicarbonate solution (10 mL each), dried over Na_2_SO_4_ and evaporated. The crude product was purified by flash chromatography (1% methanol in dichloromethane + 1.5% triethylamine, size: 18.0 × 2.0 cm, 21 g silica gel). Yield: 212 mg (40%) of compound **6** as white foam. TLC (10% methanol in dichloromethane): *R*_f_ = 0.32; ^1^H NMR (300 MHz, DMSO-*d*_6_) δ 0.89–0.96 (q, 6H, NCHN(CH_2_CH_2_CH_2_C*H*_3_)_2_), 1.28–1.37 (sextet, *J* = 6.8 Hz, 4H, NCHN(CH_2_CH_2_C*H*_2_CH_3_)_2_), 1.53–1.64 (quintet, *J* = 7.8 Hz, 4H, NCHN(CH_2_C*H*_2_CH_2_CH_3_)_2_), 3.22–3.27 (m, 2H, H-C(5’)), 3.33–3.40 (t, *J* = 7.3 Hz, 4H, NCHN(C*H*_2_CH_2_CH_2_CH_3_)_2_), 3.72 (s, 6H, 2 × OCH_3_), 4.09–4.10 (d, *J* = 4.1 Hz, 1H, H-C(4’)), 4.19–4.21 (m, 1H, H-C(3’)), 4.45–4.46 (m, 1H, H-C(2’)), 5.25–5.27 (m, 1H, OH-C(3’)), 5.60–5.62 (m, 1H, OH-C(2’)), 5.86–5.87 (d, *J* = 5.2 Hz, 1H, H-C(1’)), 6.81–6.84 (d, *J* = 6.8 Hz, 4H, H(ar)-DMTr), 7.20–7.29 (m, 8H, H(ar)-DMTr, H-C(2)), 7.34–7.36 (d, *J* = 7.4 Hz, 2H, H(ar)-DMTr), 7.78–7.80 (d, *J* = 5.6 Hz, 1H, H-C(3)), 8.24 (s, 1H, NC*H*N(CH_2_CH_2_CH_2_CH_3_)_2_), 8.64 (s, 1H, H-C(8)) ppm; ^13^C NMR (75 MHz, DMSO-*d*_6_) δ 13.61, 13.82 (NCHN(CH_2_CH_2_CH_2_*C*H_3_)_2_), 19.21, 19.73 (NCHN(CH_2_CH_2_*C*H_2_CH_3_)_2_), 28.78, 30.74 (NCHN(CH_2_*C*H_2_CH_2_CH_3_)_2_), 44.06 (C-(5')), 50.46, 52.07 (NCHN(*C*H_2_CH_2_CH_2_CH_3_)_2_), 55.01 (2 × O*C*H_3_ (DMTr)), 63.57; 70.11 (C-(3’)), 73.40 (C-2’)), 83.38 (C-4’)), 85.66, 89.00 (C-(1’)), 101.66 (C-(3)), 112.88, 113.19 (C(ar)), 126.68, 127.71, 127.83, 129.71, 133.24 (C-(ar)), 135.34, 135.39, 138.92, 140.22 (C-(2)), 140.83 (N*C*HN(CH_2_CH_2_CH_2_CH_3_)_2_), 144.73, 155.05, 155.88, 158.08 (C-(8)) ppm; HRMS [M + H^+^]: calcd for C_41_H_49_N_5_O_6_^+^, 708.3761; found, 708.3766.

***N*^6^-[(Dibutylamino)methylene]-5’-*O*-(4,4’-dimethoxytrityl)-2’-*O*-triisopropylsilyl-3-deazaadenosine (7).** Compound **6** (458 mg, 647 µmol) was weighed into a 25 mL Schlenk flask, dried in high vacuum for one hour, and dissolved in dry pyridine (3 mL) under argon atmosphere. Then, AgNO_3_ (440 mg, 2.59 mmol) was added. The reaction solution was stirred in the dark for 15 minutes, resulting in a clear solution, after which triisopropylsilyl chloride (0.55 mL, 2.59 mmol) was added. The reaction was allowed to proceed for 18 hours at room temperature. The resulting suspension was filtered through celite and partitioned between dichloromethane and 5% aqueous sodium bicarbonate solution (10 mL each). The organic layer was washed twice with 5% aqueous sodium bicarbonate solution (2 × 10 mL), dried over Na_2_SO_4_ and evaporated. The regioisomers were separated by flash chromatography (toluene/ethyl acetate/methanol = 12.5:11.5:1, size: 23.0 × 1.5 cm, 17 g silica gel). Yield: 146.2 mg (26%) of compound **7** as white foam. TLC: (10% methanol in dichloromethane): *R*_f_ = 0.47; ^1^H NMR (300 MHz, DMSO-*d*_6_) δ 0.77–0.79 (m, 6H, NCHN(CH_2_CH_2_CH_2_C*H*_3_)_2_), 0.89–0.93 (m, 18H, Si(CH(C*H*_3_)_3_)_3_), 1.23–1.35 (sextet, *J* = 6.9 Hz, 4H, NCHN(CH_2_CH_2_C*H*_2_CH_3_)_2_), 1.50–1.61 (quintet, *J* = 8.0 Hz, 4H, NCHN(CH_2_C*H*_2_CH_2_CH_3_)_2_), 3.24–3.33 (m, 7H, NCHN(C*H*_2_CH_2_CH_2_CH_3_)_2_, Si(C*H*(CH_3_)_3_)_3_), 3.49–3.54 (t, *J* = 7.3 Hz, 2H, H-C(5’)), 3.70 (s, 6H, 2 × OCH_3_), 4.10–4.13 (m, 1H, H-C(4’)), 4.19–4.24 (m, 1H, H-C(3’)), 4.67–4.71 (m, 1H, H-C(2’)), 5.20–5.22 (m, 1H, OH-C(3’)), 5.89–5.91 (d, *J* = 6.1 Hz, 1H, H-C(1’)), 6.82–6.84 (d, *J* = 7.7 Hz, 4H, H(ar)-DMTr), 7.17–7.28 (m, 8H, H(ar)-DMTr, H-C(2)), 7.34–7.37 (d, *J* = 8.0 Hz, 2H, H(ar)-DMTr), 7.63–7.65 (d, *J* = 5.3 Hz, 1H, H-C(3)), 8.26 (s, 1H, NC*H*N(CH_2_CH_2_CH_2_CH_3_)_2_), 8.59 (s, 1H, H-C(8)) ppm; ^13^C NMR (150 MHz, CDCl_3_) δ 12.01, 13.77, 14.02 (Si(CH(*C*H_3_)_3_)_3_), 17.49, 17.67 (NCHN(CH_2_CH_2_CH_2_*C*H_3_)_2_), 19.88, 20.27 (NCHN(CH_2_CH_2_*C*H_2_CH_3_)_2_), 29.30, 31.22 (NCHN(CH_2_*C*H_2_CH_2_CH_3_)_2_), 44.82 (NCHN(*C*H_2_CH_2_CH_2_CH_3_)_2_), 51.37 (Si(*C*H(CH_3_)_3_)_3_), 55.23, 63.51 (C-(5’)), 71.75 (C-(3’)), 75.57 (C-(2’)), 83.97 (C-(4’)), 86.99, 89.17 (C-(1’)), 101.57 (C-(2)), 113.26, 127.05, 127.97, 128.16, 130.09, 130.16 (C(ar)-DMTr), 133.63, 135.29, 135.38, 139.03, 140.49 (N*C*HN(CH_2_CH_2_CH_2_CH_3_)_2_), 141.42 (C-(3)), 144.37, 155.87, 156.29 (C-(8)), 158.63 ppm; HRMS [M + H^+^]: calcd for C_50_H_69_N_5_O_6_Si^+^, 864.5095; found, 864.5149.

***N*^6^-[(Dibutylamino)methylene]-5’-*O*-(4,4’-dimethoxytrityl)-2’-*O*-triisopropylsilyl-3-deazaadenosine 3’-(2-cyanoethyl diisopropylphosphoramidite) (8).** Compound **7** (124 mg, 155 µmol) was weighed into a 5 mL flask, dried in high vacuum for 1 hour and dissolved in dry dichloromethane (1 mL) under argon atmosphere. Then, *N,N-*dimethylethylamine (170 µL, 1.55 mmol) was added and the reaction mixture was stirred for 15 minutes at room temperature. 2-Cyanoethyl-*N*,*N*-diisopropylchlorophosphoramidite (110 µL, 466 µmol) was added and the reaction was allowed to proceed for 90 minutes, after which it was quenched by the addition of methanol (1 mL). The residue was diluted with dichloromethane (10 mL) and washed with saturated sodium bicarbonate solution (10 mL). The organic layer was then dried over Na_2_SO_4_ and evaporated, and the resulting crude product was purified by flash chromatography (50% ethyl acetate in cyclohexane, size: 19.0 × 1.5 cm, 15 g silica gel). Yield: 73 mg (44%) of compound **8** as white foam. TLC (50% ethyl acetate in cyclohexane): *R*_f_ = 0.56; ^1^H NMR (300 MHz, DMSO-*d*_6_) δ 0.74–0.95 (m, 48H, NCHN(CH_2_CH_2_CH_2_C*H*_3_)_2_^a,b^), Si(CH(C*H*_3_)_3_)_3_^a,b^), 1.12–1.23 (m, 24H, N(CH(C*H*_3_)_2_)_2_^a,b^), 1.28–1.36 (m, 8H, NCHN(CH_2_CH_2_C*H*_2_CH_3_)_2_^a,b^), 1.55–1.60 (m, 8H, NCHN(CH_2_C*H*_2_CH_2_CH_3_)_2_^a,b^), 2.75–2.82 (m, 4H, CH_2_C*H*_2_CN^a,b^), 3.29–3.35 (m, 12H, H-C(5’)^a,b^, NCHN(C*H*_2_CH_2_CH_2_CH_3_)_2_^a,b^), 3.49–3.65 (m, 14H, H-C(4’)^a,b^, Si(C*H*(CH_3_)_3_)_3_^a,b^, N(C*H*(CH_3_)_2_)_2_^a,b^), 3.79–3.90 (m, 4H, C*H*_2_CH_2_CN^a,b^), 4.25–4.29 (m, 1H, H-C(3’)^b^), 4.37–4.43 (m, 1H, H-C(3’)^a^), 4.84–4.93 (m, 2H, H-C(2’)^a,b^), 5.82–5.87 (d, *J* = 7.0 Hz, 1H, H-C(1’)^b^), 5.93–5.95 (d, *J* = 7.0 Hz, 1H, H-C(1’)^a^), 6.85–6.87 (d, *J* = 7.7 Hz, 8H, H(ar)-DMTr^a,b^), 7.19–7.32 (m, 16H, H(ar)-DMTr^a,b^, H-C(2)^a,b^), 7.37–7.39 (d, *J* = 6.3 Hz, 4H, H(ar)-DMTr^a,b^), 7.60–7.64 (m, 2H, H-C(3)^a,b^), 8.28 (s, 2H, NC*H*N(CH_2_CH_2_CH_2_CH_3_)_2_^a,b^), 8.61 (s, 2H, H-C(8)^a,b^) ppm; ^31^P (121 MHz, CDCl_3_) δ 149.52, 153.84 ppm; HRMS [M + H^+^]: calcd for C_59_H_87_N_7_O_7_PSi^+^, 1064.6168; found, 1064.6192.

## Supporting Information

File 1 Synthetic procedures of compounds **1**–**3** and NMR spectra of compounds **1**–**8**.
