# Supplementary material for: Facile synthesis of a 3-deazaadenosine phosphoramidite for RNA solid-phase synthesis
Source: Beilstein J Org Chem. 2016 Nov 28;12:2556–62. doi: 10.3762/bjoc.12.250 (PMC5238537; doi:10.3762/bjoc.12.250)
Supplement: File 1 — Synthetic procedures of compounds 1–3 and NMR spectra of compounds 1–8. [file Beilstein_J_Org_Chem-12-2556-s001.pdf]

Supporting Information

for

Facile synthesis of a 3-deazaadenosine phosphoramidite for RNA solid-phase synthesis

Elisabeth Mairhofer[‡,1], Elisabeth Fuchs[‡,1] and Ronald Micura*[1]

Address: [1]Institute of Organic Chemistry and Center for Molecular Biosciences, University of Innsbruck, Austria

Email: Ronald Micura - ronald.micura@uibk.ac.at

* Corresponding author

‡ Equal contributors

Synthetic procedures of compounds 1–3 and NMR spectra of compounds 1–8

Contents

1. Synthesis of tribenzoylated 6-azido-3-deazapurine nucleoside (**2**) S2
  1.1. 6-Azido-3-deazapurine (**1**) S2
  1.2. 6-Azido-3-deazapurine ribonucleoside (**2**) S3
2. Synthesis of 6-amino-3-deazapurine (**3**) S4
3. NMR spectra of compounds **4** to **8** S6
  3.1. 2',3',5'-Tri-*O*-benzoyl-3-deazaadenosine (**4**) S6
  3.2. 3-Deazaadeonsine (**5**) S7
  3.3. *N*[6]-[(Dibutylamino)methylene]-5’-*O*-(4,4’-dimethoxytrityl)-3-deazaadenosine (**6**) S8
  3.4. *N*[6]-[(Dibutylamino)methylene]-5’-*O*-(4,4’-dimethoxytrityl)-2’-*O*-triisopropylsilyl-3-deazaadenosine (**7**) S9
  3.5. *N*[6]-[(Dibutylamino)methylene]-5’-*O*-(4,4’-dimethoxytrityl)-2’-*O*-triisopropylsilyl-3-deazaadenosine 3’-(2-cyanoethyl diisopropylphosphoramidite) (**8**) S10
4. ROESY NMR spectrum of compound **4** S11
5. HMBC NMR spectrum of compound **5** S12
6. Comparison of ^{1}H NMR spectra of compound **5** S13

1. Synthesis of tribenzoylated 6-azido-3-deazapurine nucleoside (2)

1.1. 6-Azido-3-deazapurine (1)

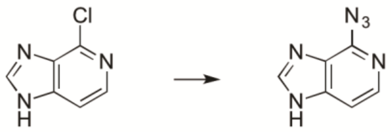

1

6-Chloro-3-deazapurine (207 mg, 1.35 mmol) and 85 mg (1.74 mmol) LiN_3 were dissolved in *N*,*N*-dimethylformamide (4 mL) and stirred for 18 h at 120 °C. The solvents were evaporated, the residue was suspended in cold water and filtered over a glass sintered funnel. The precipitate was washed with cold water and dried overnight on high vacuum. Yield: 164 mg (76%) of compound **1** as beige powder. TLC: (CH_2Cl_2 /MeOH, 9/1): R_f = 0.38; ^{1}H-NMR (300 MHz, DMSO-d_6): δ 7.69 (d, 1H, J = 7.3 Hz, H-C(3)); 8.54 (s, 1H, H-C(8)); 9.05 (d, 1H, J = 7.3 Hz, H-C(2)) ppm; ^{13}C-NMR (75 MHz, d_6-DMSO): δ 106.46 (C(3)); 120.62 (C(2)); 125.06; 134.59; 143.38 (C(8)); 143.55 ppm; FT-ICR-MS *(m/z)*: $[M+H]^+$ calcd for $C_6H_4N_6$ 161.06; found 161.06.

^{1}H NMR (300 MHz, DMSO-d_6) spectrum of compound **1:**

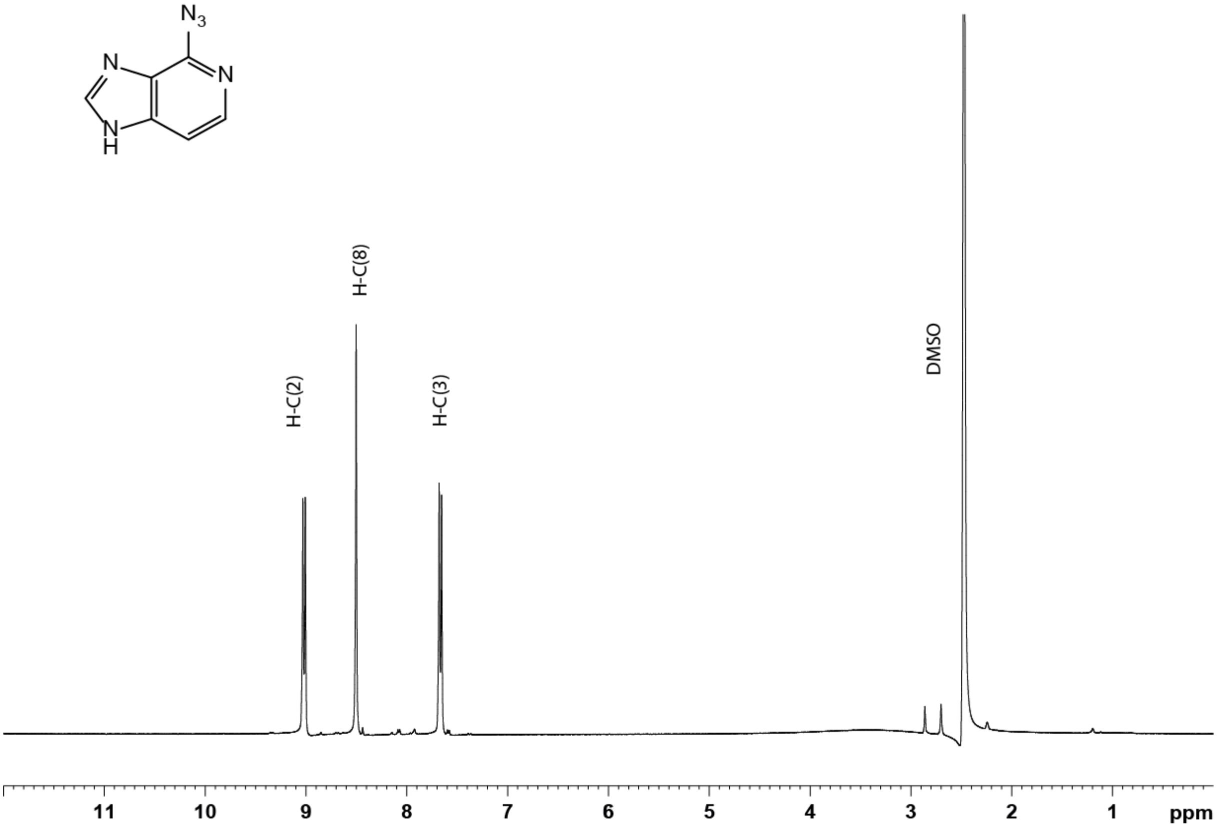

1.2. 1-(2’,3’,5’-Tri-*O*-benzoyl-ß-D-ribofuranosyl)-6-azido-3-deazapurine (2)

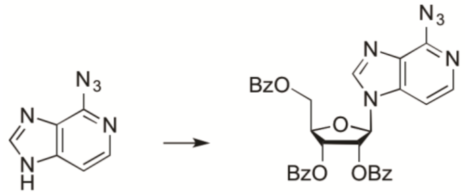

1 → 2

1-*O*-Acetyl-2,3,5-tri-*O*-benzoyl-*β*-D-ribofuranose (315 mg, 0.62 mmol) and 6-azido-3-deazapurine (**1**, 97 mg, 0.62 mmol) were suspended in toluene (50 mL) before *N*,*O*-bis(trimethylsilyl)acetamide (510 µL, 2.09 mmol) was added at room temperature. The suspension was heated up to 105 °C, stirred under argon for 30 min. To the clear solution, trimethylsilyl trifluoromethanesulfonate (340 µL, 1.88 mmol) was added and the reaction mixture was stirred for 40 min at 105 °C. Then, all solvents were evaporated, the residue was dissolved in CH_2Cl_2, washed twice with saturated sodium bicarbonate solution and dried over Na_2SO_4. The crude product was purified by column chromatography on SiO_2 (1% to 5% acetone in CH_2Cl_2, v/v). Yield: 268 mg (71%) of compound **2** as white foam. TLC: (CH_2Cl_2 /acetone 95/5): R_f = 0.39; ^{1}H-NMR (300 MHz, $CDCl_3$): δ 4.89- 5.03 (m, 3H, H-C(5’)), H-C(5’’)), H-C(4’)); 6.19- 6.20 (m, 2H, H-(C2’)), H-C(3’)); 6.98 (d, J = 5.2 Hz, 1H, H-C(1’)); 7.27 - 7.62 (m, 10H, H- C(benzoyl)), H-C(3)); 7.86-8.12 (3d, 6H, H-C(benzoyl)); 8.36 (s, 1H, H-C(8)); 8.68 (d, J = 7.3, 1H, H-C(2)) ppm; ^{13}C-NMR (75 MHz, $CDCl_3$): δ 63.89 (C(5’)); 71.39 (C(2’)); 75.05 (C(3’)); 81.43 (C(4’)); 89.16 (C(1‘)); 111.54 (C(3)); 117.77; 120.82 (C(2)); 128.11; 128.53, 128.65, 128.73, 129.33, 129.77, 129.93, 133.57, 133.87, (C(benzoyl)); 140.98; 142.79 (C(8)); 143.73; 165.10; 165.39; 166.17 ppm; ESI-MS *(m/z)*: $[M+H]^+$ calcd for $C_{32}H_{23}N_6O_7$ 605.18; found 604.80.

^{1}H NMR (300 MHz, $CDCl_3$) spectrum of compound **2**:

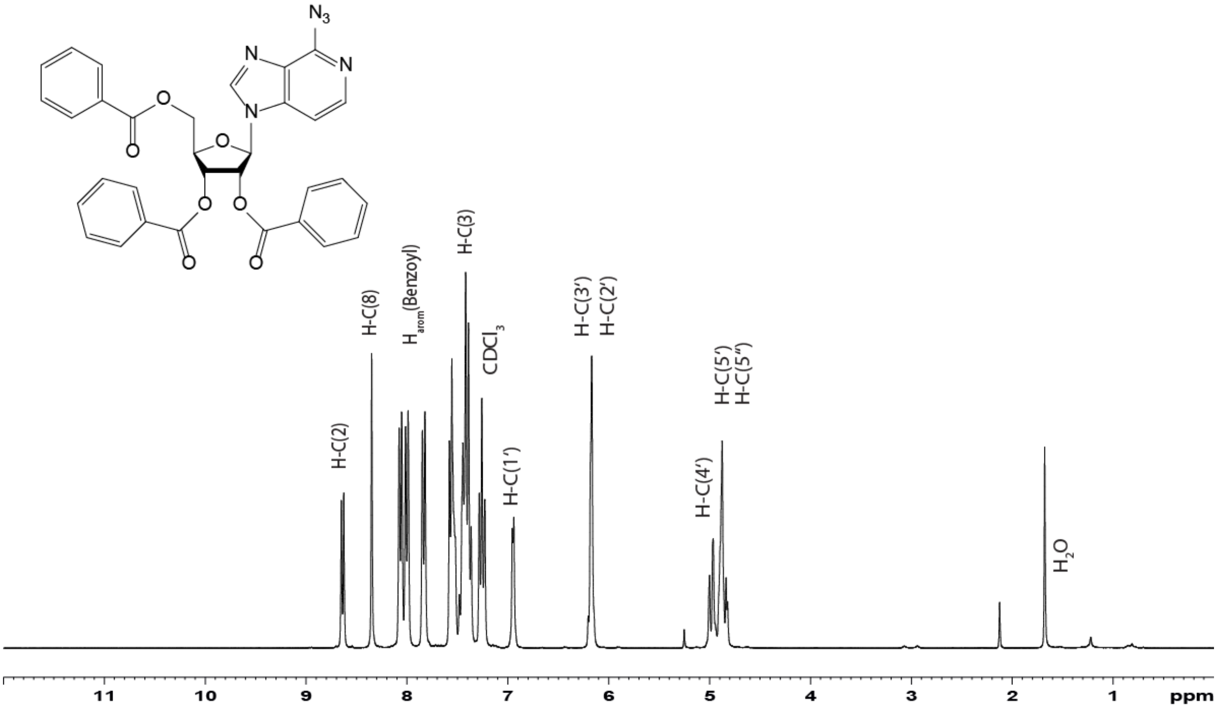

2. Synthesis of 6-amino-3-deazapurine (3)

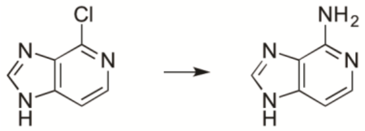

3

Procedure A: 6-Chloro-3-deazapurine (303 mg, 5.80 mmol) was dissolved in 1,4-dioxane (6 mL) and aqueous ammonia (6 mL, 32%). Using a microwave reactor, the reaction solution was kept at 140 °C for 2 h, followed by evaporation of all volatiles. The crude product was purified by flash chromatography (2 to 10% methanol in dichloromethane; + 1.5% triethylamine, size: 21.0 × 2.5 cm, 28 g silica gel). Yield: 239 mg (90%) of compound **3** as slightly yellow powder.

Procedure B: 6-Chloro-3-deazapurine (890 mg, 5.80mmol) was dissolved in 1,4 dioxane (18 mL) and 32% (aq) ammonia (18 mL). The solution was transferred into three glass tubes à 12 mL, the tubes were sealed and heated to 180 °C for 74 h. The solvents were evaporated, the residue was dissolved in boiling ethanol, and then allowed to cool down at room temperature. The white salt precipitation was filtered, the filtrate was evaporated again and purified by column chromatography on SiO_2 (2 to 10% methanol in dichlormethane; + 5% triethylamine).

TLC: (15% methanol in dichloromethane): R_f = 0.42. ^{1}H-NMR (300 MHz, DMSO-d_6): δ = 6.07 (s, br, 2H, NH_2), 6.73-6.75 (d, J = 5.7 Hz, 1H, H-C(3)), 7.60-7.62 (d, J = 5.7 Hz, 1H, H-C(2)), 8.06 (s, 1H, H-C(8)) ppm. ^{13}C-NMR (75 MHz, DMSO-d_6): δ = 98.96 (C(3)); 124.84; 139.30 (C(2)); 139.65; 139.80 (C(8)); 151.18 ppm. HR-MS $[M+H]^+$: calcd for $C_6H_6N_4^+$ 135.0665; found 135.0676.

^{1}H NMR (300 MHz, DMSO-d_6) spectrum of compound **3**:

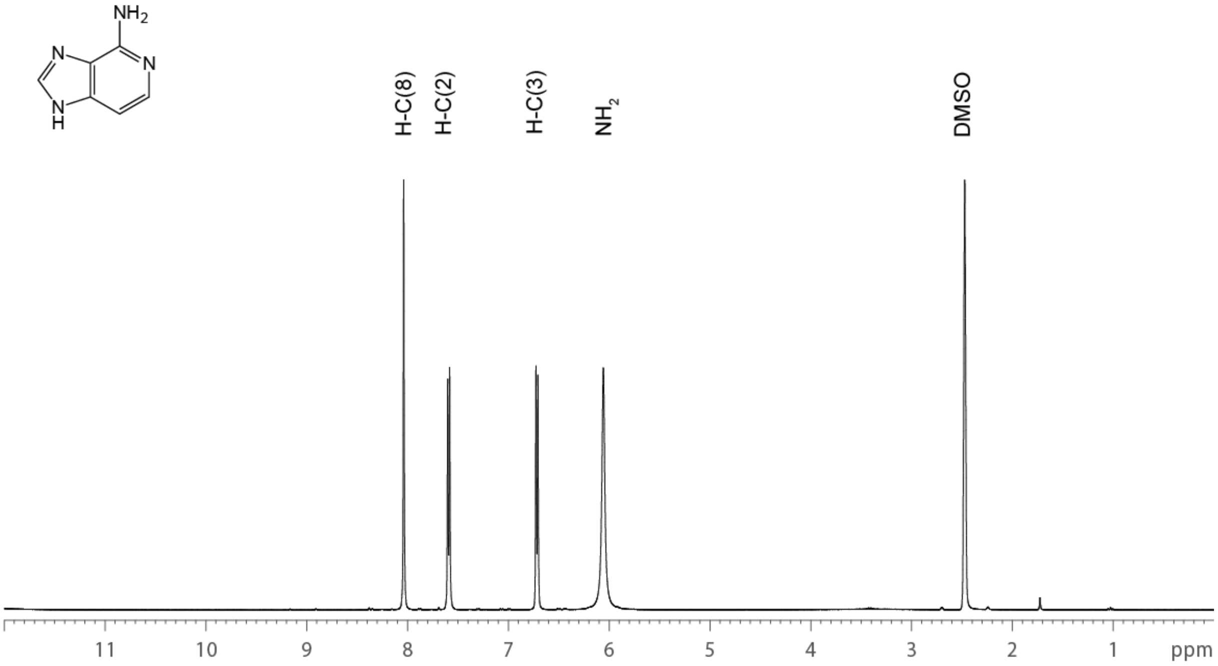

^{13}C NMR (75 MHz, DMSO-d_6) spectrum of compound **3**:

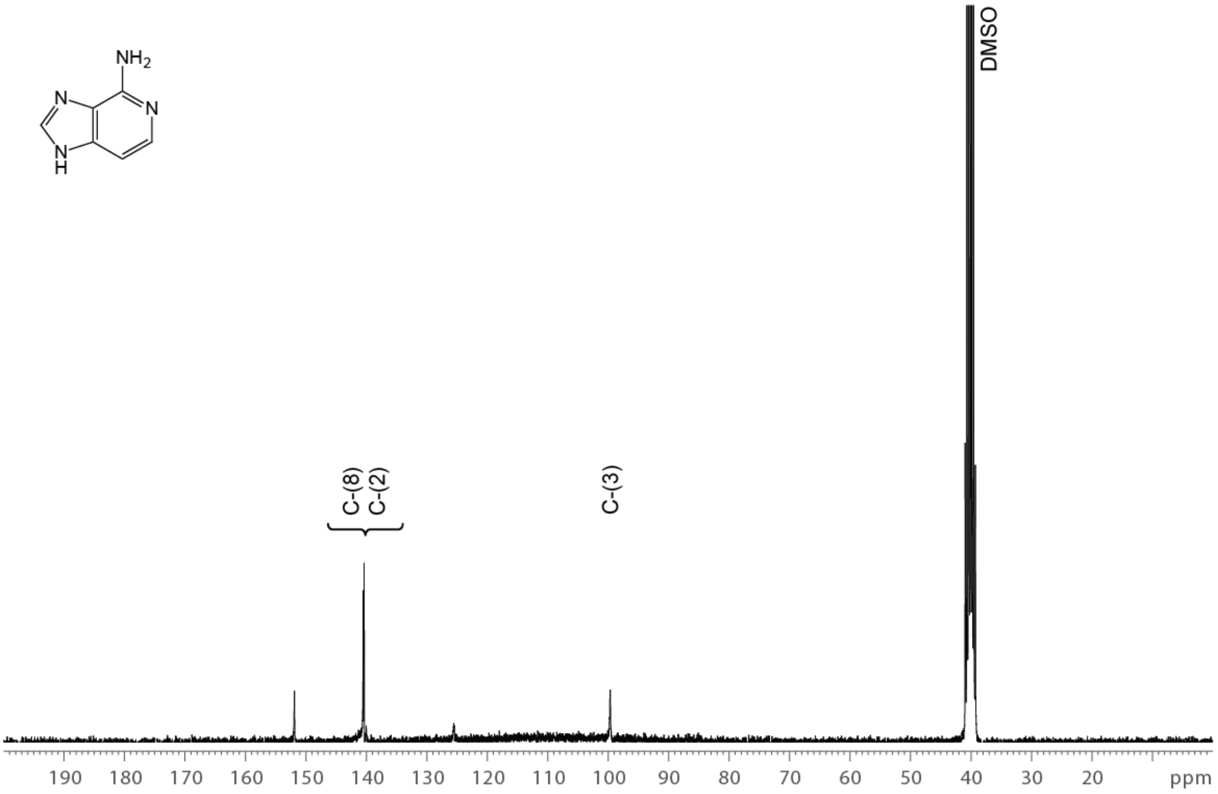

3. NMR spectra of compounds 4 to 8

3.1. 2',3',5'-Tri-*O*-benzoyl-3-deazaadenosine (4)

^{1}H NMR (300 MHz, DMSO-d_6) spectrum of compound **4**:

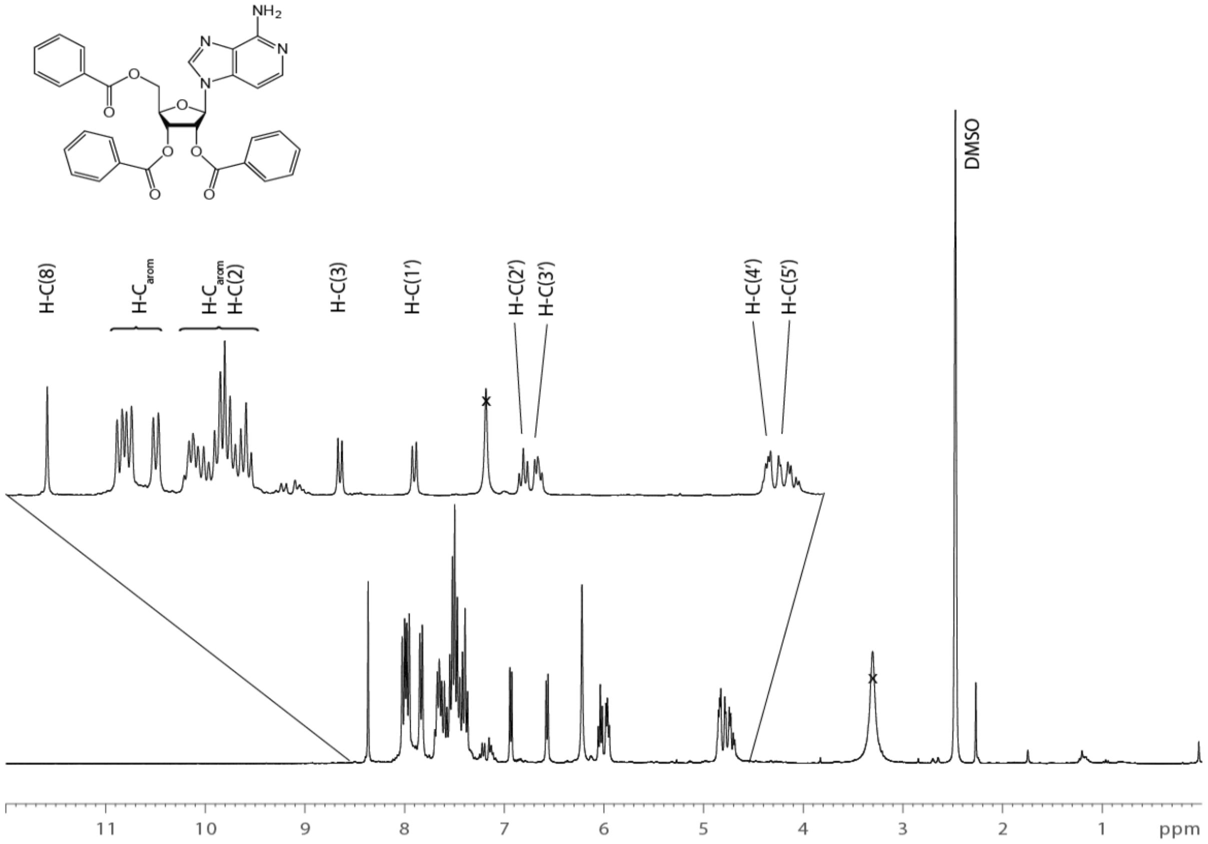

^{13}C NMR (75 MHz, DMSO-d_6) spectrum of compound **4**:

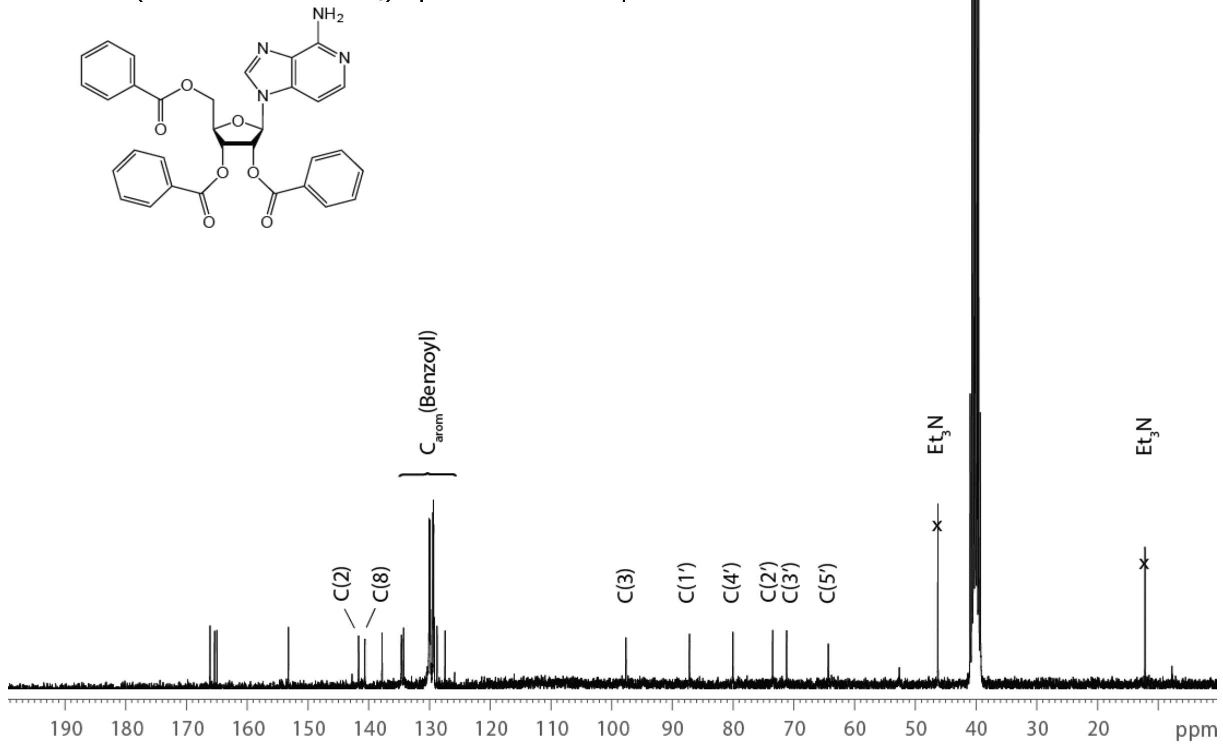

3.2. 3-Deazaadeonsine (5)

^{1}H NMR (300 MHz, DMSO-d_6) spectrum of compound **5**:

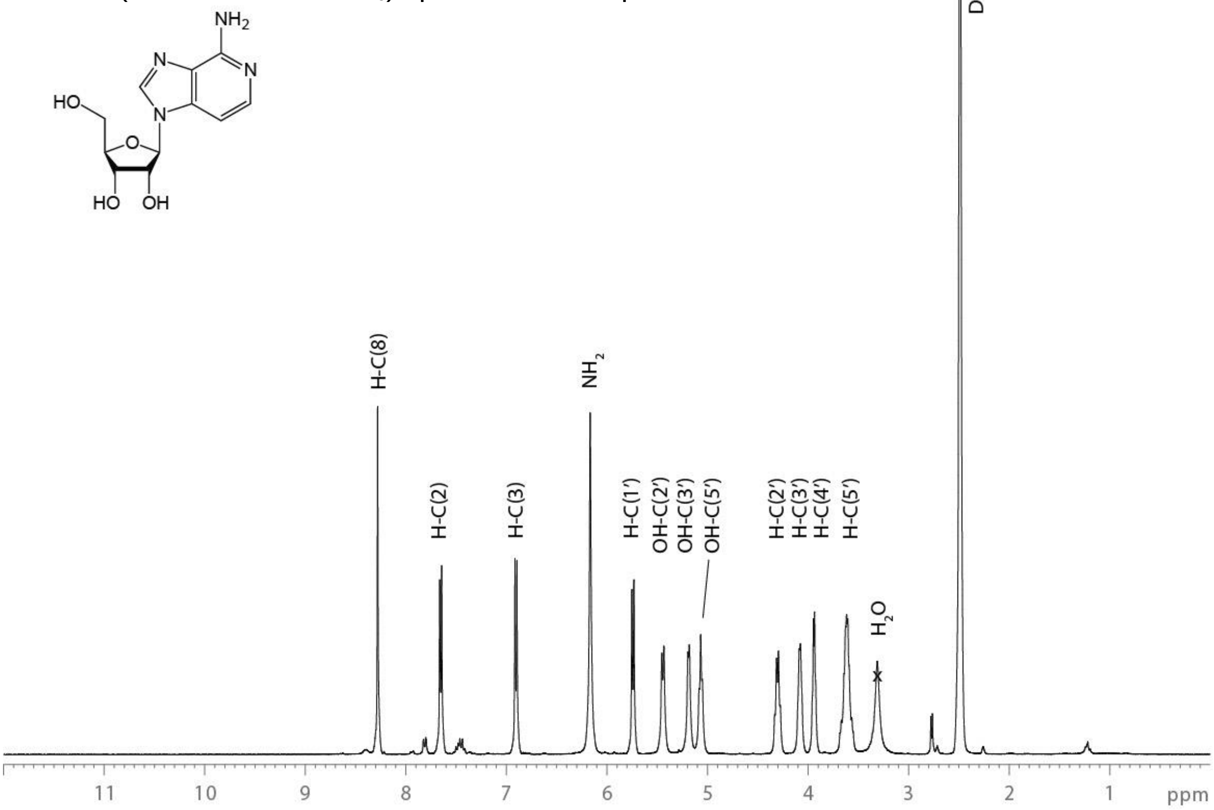

^{13}C NMR (75 MHz, DMSO-d_6) spectrum of compound **5**:

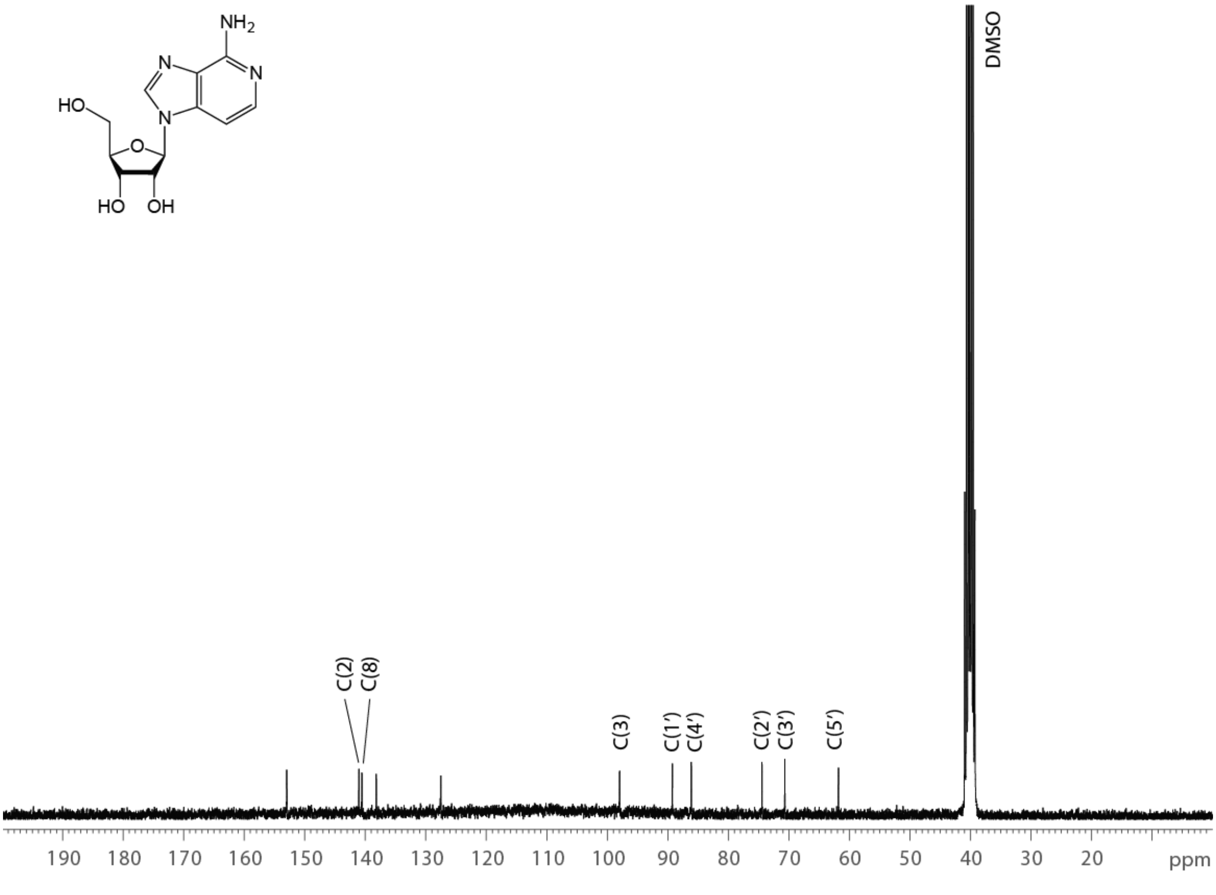

3.3. *N*[6]-[(Dibutylamino)methylene]-5'-*O*-(4,4'-dimethoxytrityl)-3-deazaadenosine (6)

^{1}H NMR (300 MHz, DMSO-d_6) spectrum of compound **6**:

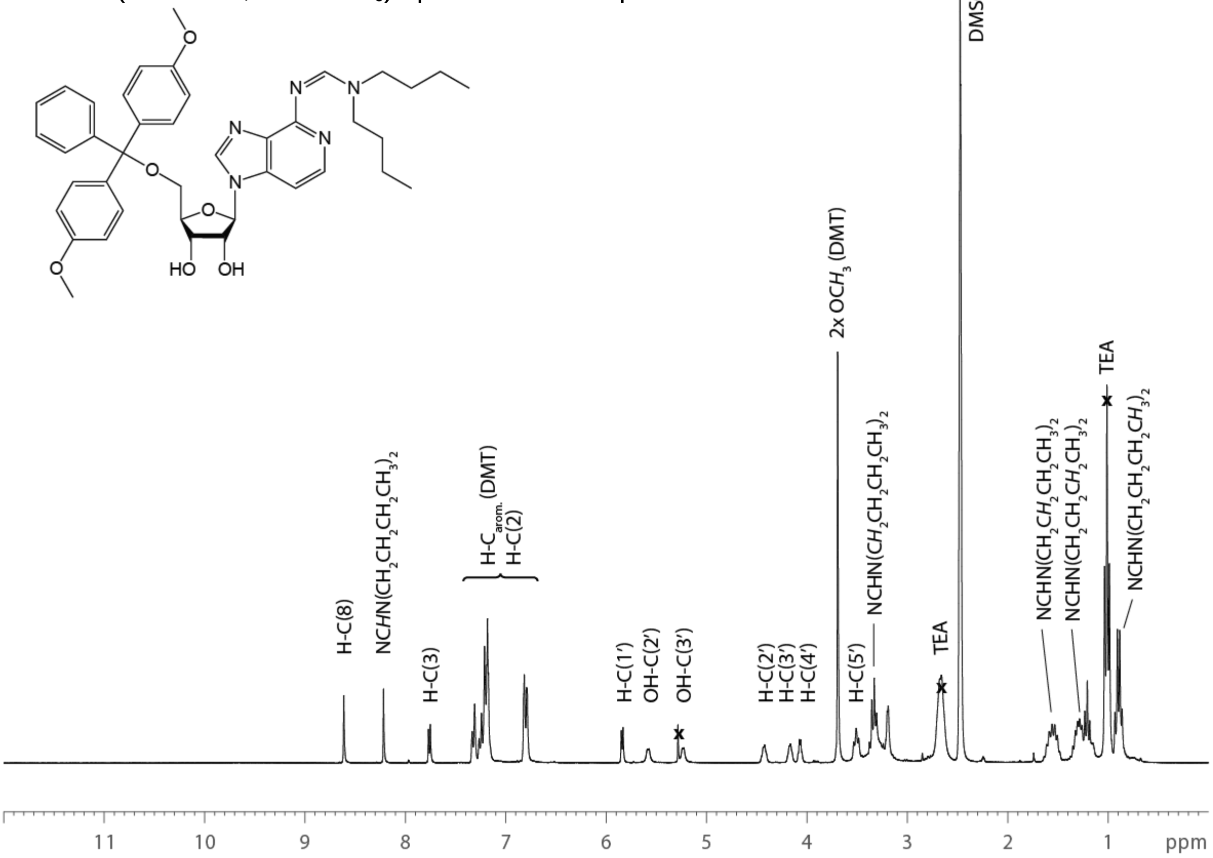

^{13}C NMR (75 MHz, DMSO-d_6) spectrum of compound **6**:

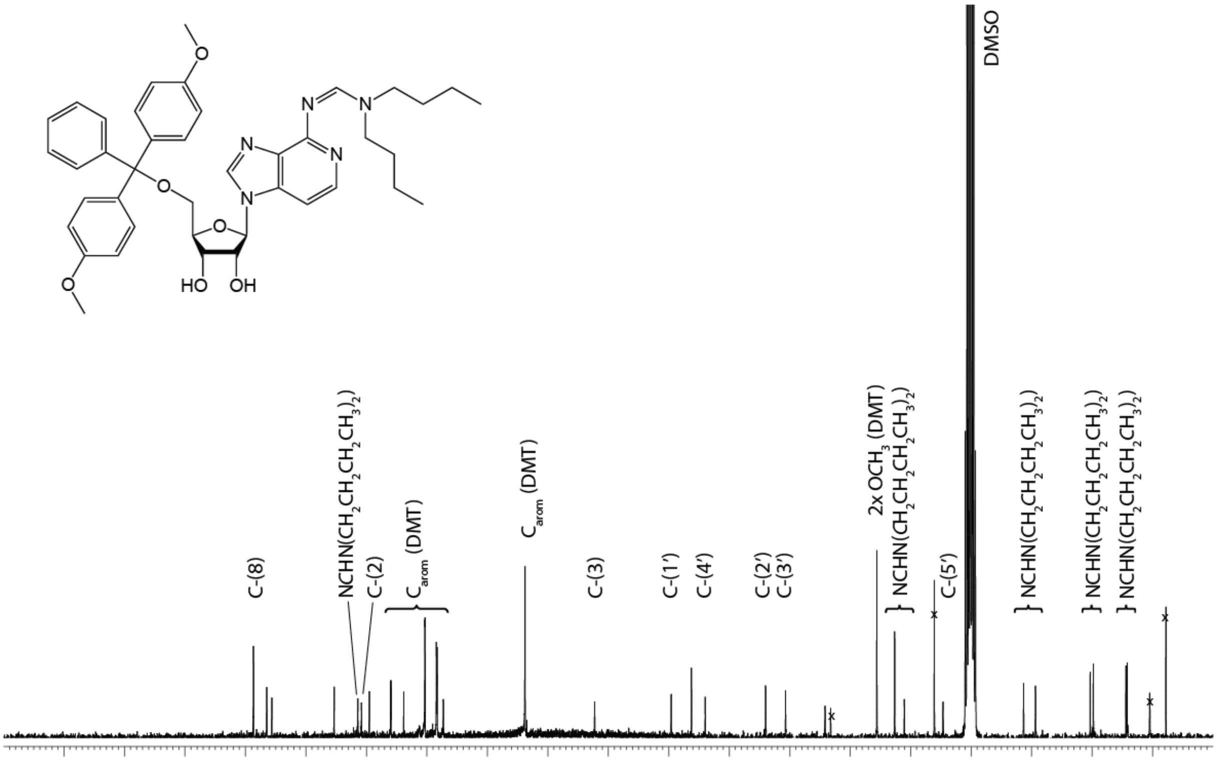

3.4. *N*[6]-[(Dibutylamino)methylene]-5'-*O*-(4,4'-dimethoxytrityl)-2'-*O*-triisopropylsilyl-3-deazaadenosine (7)

^{1}H NMR (300 MHz, DMSO-d_6) spectrum of compound **7**:

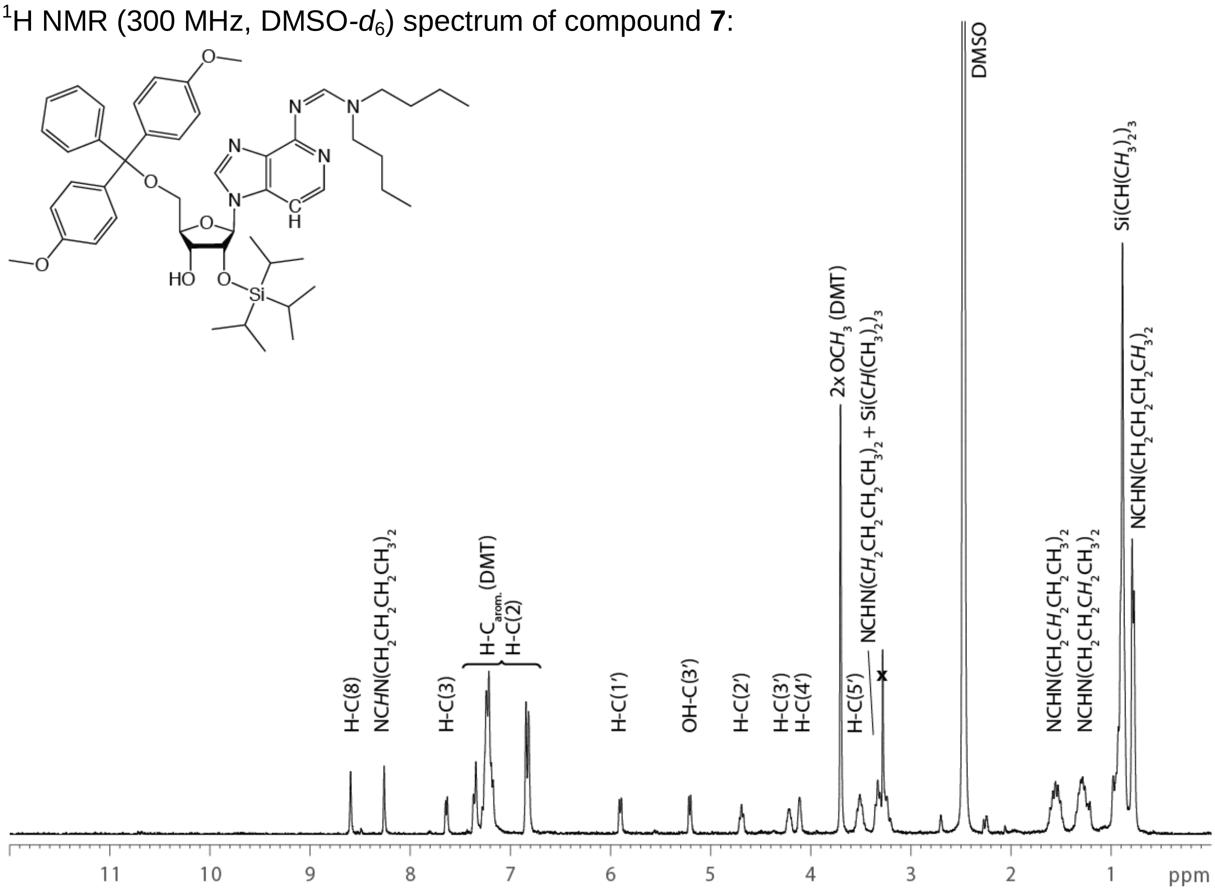

^{13}C NMR (150 MHz, $CDCl_3$) spectrum of compound **7**:

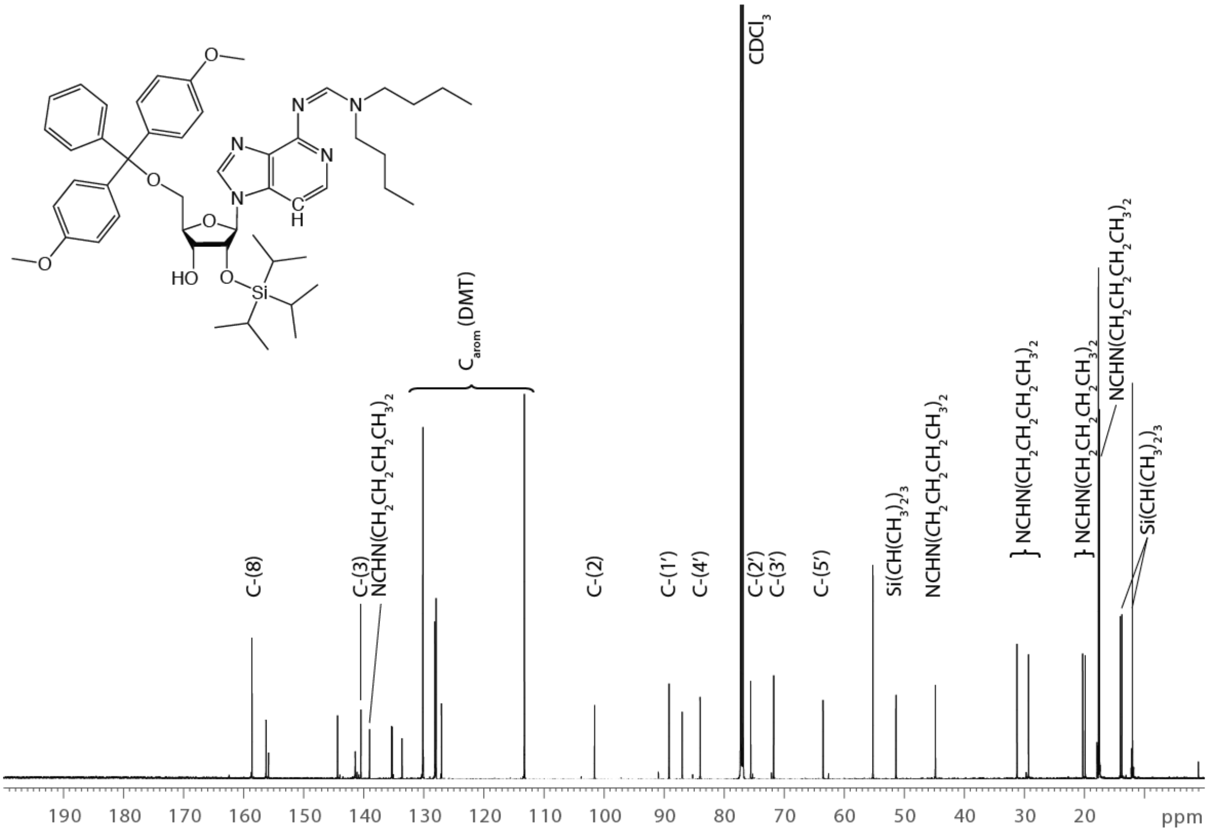

3.5. N^6-[(Dibutylamino)methylene]-5'-*O*-(4,4'-dimethoxytrityl)-2'-*O*-triisopropylsilyl-3-deazaadenosine 3'-(2-cyanoethyl diisopropylphosphoramidite) (8)

^{1}H NMR (300 MHz, DMSO-d_6) spectrum of compound **8**:

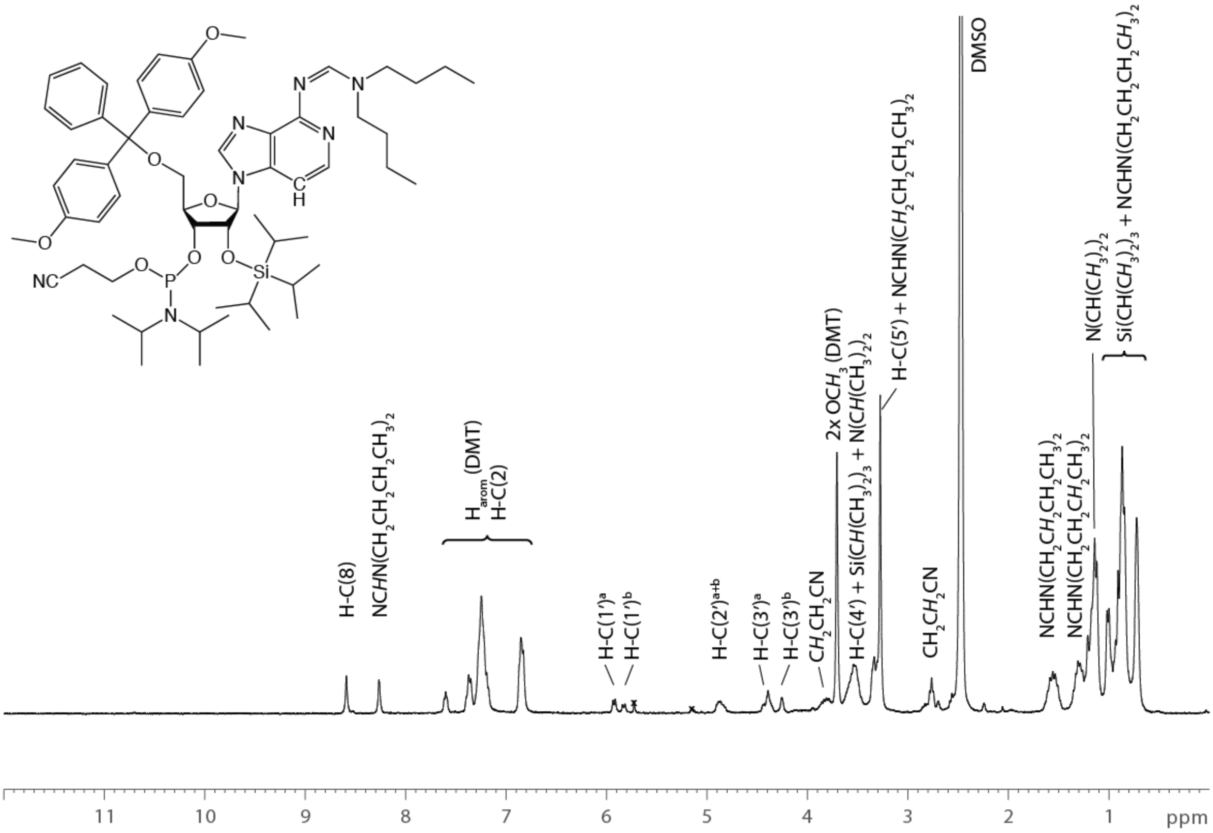

^{31}P NMR (121 MHz, CDCl$_3$) spectrum of compound **8**:

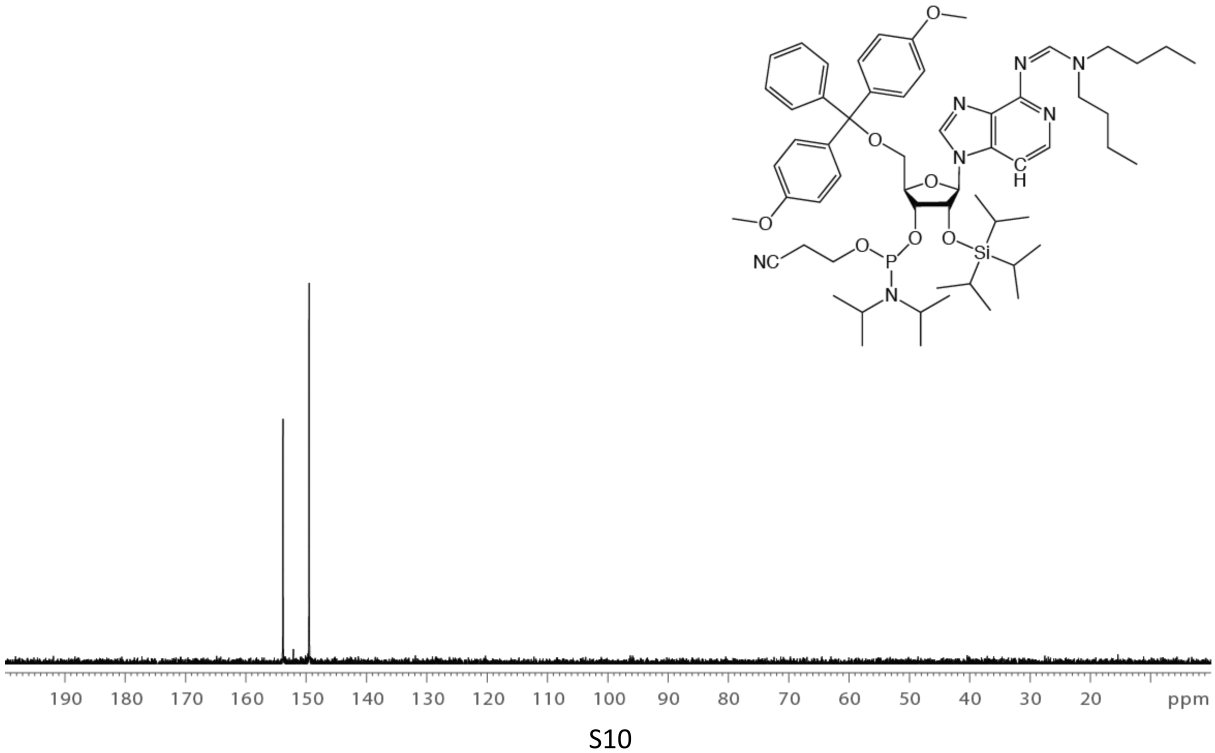

4. ROESY NMR spectrum of compound 4 (500 MHz, DMSO-d_6)

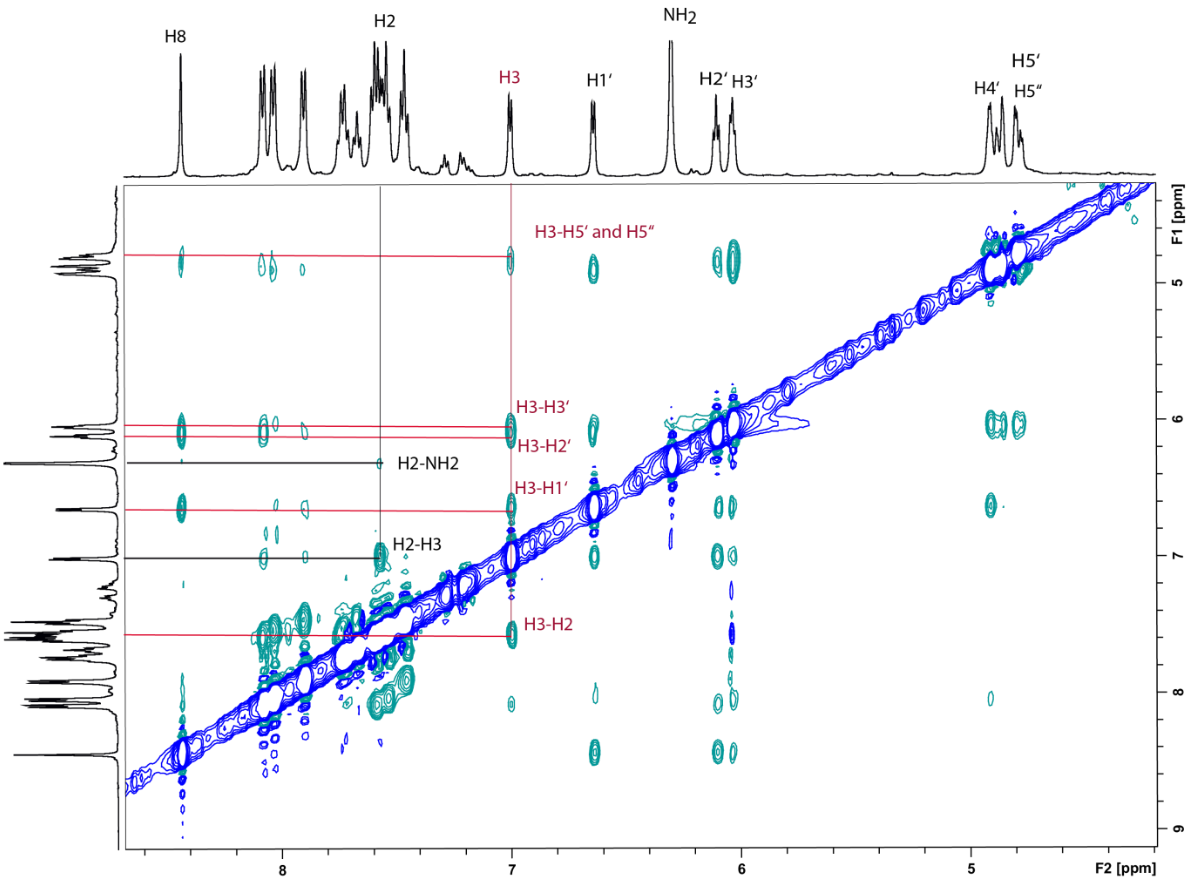

5. HMBC NMR spectrum of compound 5 (600 MHz, DMSO-d_6)

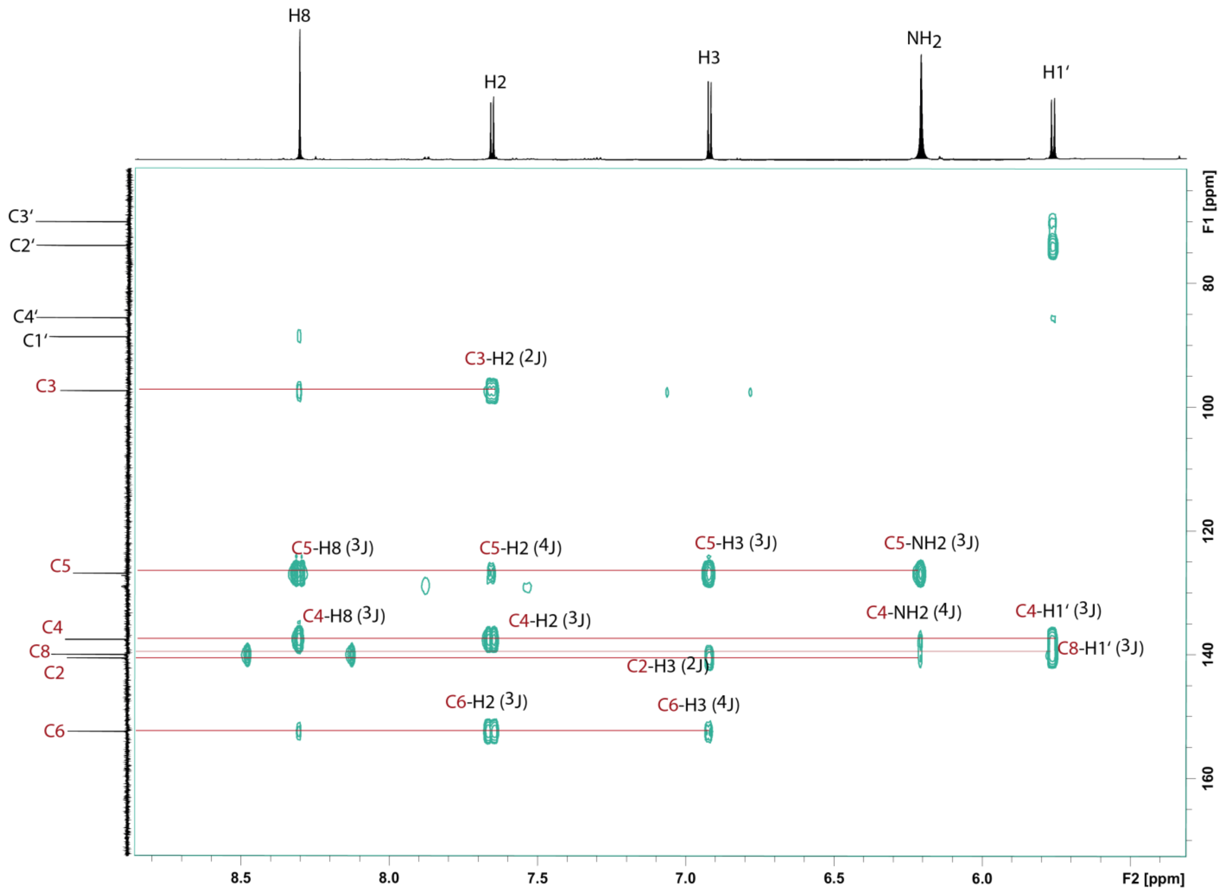

6. Comparison of ^{1}H NMR spectra of compound 5

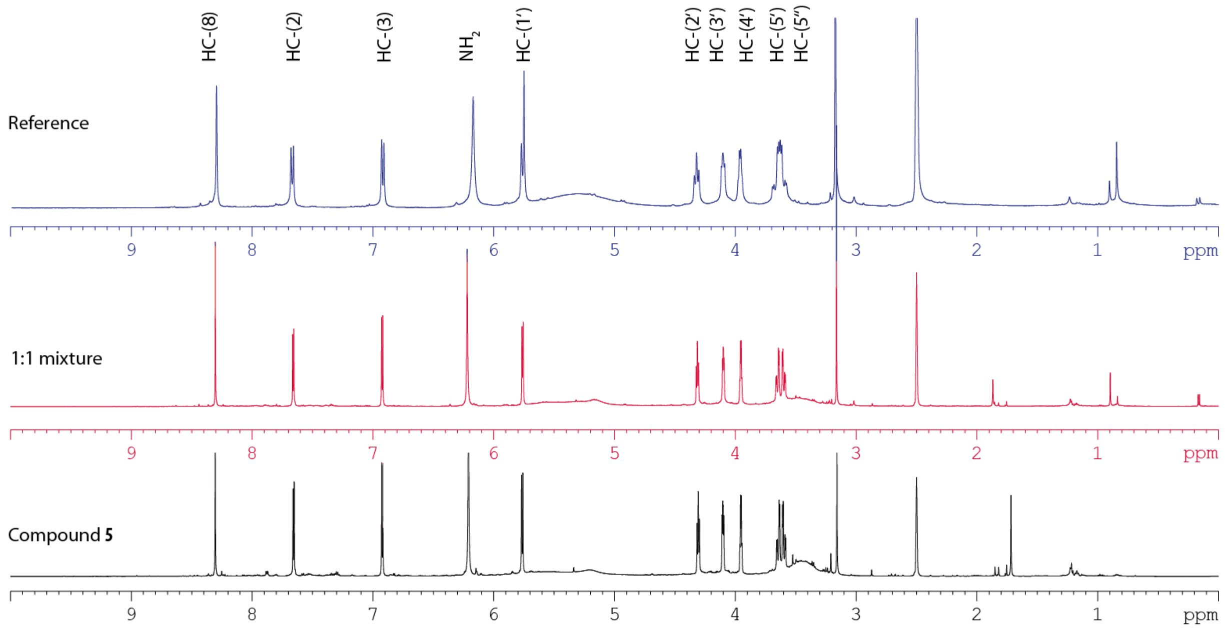

Comparison of ^{1}H-NMR (600 MHz, DMSO-d_6) spectra of 3-deazaadenosine **5** (synthesized according to the pathway given in this paper) with 3-deazaadenosine reference sample that was synthesized from inosine *via* a previously published 12-step route [Erlacher, M. D., Lang, K., Wotzel, B., Rieder, R., Micura, R., & Polacek, N. (2006). Efficient Ribosomal Peptidyl Transfer Critically Relies on the Presence of the Ribose 2‘-OH at A2451 of 23S rRNA. *Journal of the American Chemical Society*, *128*(13), 4453–4459]. Top: 3-deazaadenosine reference sample; middle: 1:1 mixture of 3-deazaadenosine **5** and 3-deazaadenosine reference sample; bottom: 3-deazaadenosine **5**.
